# Alcohol consumption in relation to maternal deaths from induced-abortions in Ghana

**DOI:** 10.1186/1742-4755-9-10

**Published:** 2012-08-06

**Authors:** Benedict O Asamoah, Anette Agardh

**Affiliations:** 1 Social Medicine and Global Health, Department of Clinical Sciences, Malmö, Lund University, Lund, Sweden; 2 Centre for Adolescent Health, Murdoch Children's Research Institute, Royal Children's Hospital, University of Melbourne, Victoria, Australia

**Keywords:** Alcohol consumption, Unwanted pregnancies, Maternal mortality, Induced-abortion, Ghana

## Abstract

**Introduction:**

The fight against maternal deaths has gained attention as the target date for Millennium Development Goal 5 approaches. Induced-abortion is one of the leading causes of maternal deaths in developing countries which hamper this effort. In Ghana, alcohol consumption and unwanted pregnancies are on the ascendancy. We examined the association between alcohol consumption and maternal mortality from induced-abortion. We further analyzed the factors that lie behind the alcohol consumption patterns in the study population.

**Method:**

The data we used was extracted from the Ghana Maternal Health Survey 2007. This was a national survey conducted across the 10 administrative regions of Ghana. The survey identified 4203 female deaths through verbal autopsy, among which 605 were maternal deaths in the 12 to 49 year-old age group. Analysis was done using Statistical software IBM SPSS Statistics 20. A case control study design was used. Cross-tabulations and logistic regression models were used to investigate associations between the different variables.

**Results:**

Alcohol consumption was significantly associated with abortion-related maternal deaths. Women who had ever consumed alcohol (OR _adjusted_ 2.6, 95% CI 1.38–4.87), frequent consumers (OR _adjusted_ 2.6, 95% CI 0.89–7.40) and occasional consumers (OR _adjusted_ 2.7, 95% CI 1.29–5.46) were about three times as likely to die from abortion-related causes compared to those who abstained from alcohol. Maternal age, marital status and educational level were found to have a confounding effect on the observed association.

**Conclusion:**

Policy actions directed toward reducing abortion-related deaths should consider alcohol consumption, especially among younger women. Policy makers in Ghana should consider increasing the legal age for alcohol consumption. We suggest that information on the health risks posed by alcohol and abortion be disseminated to communities in the informal sector where vulnerable groups can best be reached.

## Introduction

The fight against maternal deaths is continually gaining attention [1-3], especially as recent multi-country studies on the attainment of Millennium Development Goal 5 (MDG 5) have shown that, for most countries, this MDG may be a mirage [4,5]. A Study by Hogan and colleagues in 2010 found that only 23 of 181 countries were on track to achieve a 75% reduction in maternal mortality ratio by the year 2015 [4]. An updated analysis a year later by Lozano and colleagues concluded that that number was only 13 [5]. Whereas the Hogan report [4] had recommended policy actions aimed at interventions in the case of the most vulnerable women in all countries, Lozano and colleagues, while supporting this, stressed on the equitable distribution of resources to accelerate countries’ progression towards the attainment of MDG 5 [5]. Unsafe abortion is one of the leading causes of maternal morbidity and mortality Worldwide [6-8]. WHO defines unsafe abortion as the termination of unintended pregnancy that is carried out by persons lacking the necessary skills or in an environment that does not meet minimum medical standards, or both [7]. Globally, abortion rate declined from 35 abortions per 1000 women aged 15–44 years in 1995 to 29 per 1000 women in 2003 and has since stalled between 2003 and 2008 (29 abortions per 1000 women in 2008) [9]. In 2008 alone, about 6 million abortions were reported in developed countries and 38 million in developing countries. It was reported in the same year that 97% of abortions in Africa were unsafe [10]. Almost all abortion related deaths occur in developing countries mostly in Africa. Despite these alarming statistics, there is evidence that induced abortion could be a safe medical procedure when conducted under the right conditions [11,12].

Hogan and colleagues reported that in 2008 more than half of the maternal deaths worldwide occurred in only six countries: India, Nigeria, Afghanistan, Ethiopia, and the Democratic Republic of the Congo. In both studies, the country level analysis included no investigations of socio-demographic subgroups and at-risk populations. While these large aggregated studies are valuable for evaluating interim achievements with regard to MDG 5, their utility in identifying the most vulnerable population subgroups is minimal [13]. For example, Hogan and colleagues recommended focusing on women, a particularly exposed population [4], considering this an ethical imperative and a matter of social justice [14]; but more research is needed to identify high risk groups in order to address equity gaps. Targeted studies that have led to reducing cause-specific maternal mortality in at-risk populations are limited. The present investigation has been motivated by the absence of policy measures that adequately deal with the needs of different socio-demographic subgroups in the hope that renewed attention may facilitate the attainment of MDG 5 in Ghana.

In Ghana, maternal mortality remains high [5,15] despite positive sustained economic growth over the past two decades [16]. According to the World Health Statistics[17], Ghana reported 630 maternal deaths per 100,000 live births in 1990 (range 340–1200), and 350 (range 210–600) in 2008 (12). Hogan and colleagues estimated 549 (range 444–1157) and 409 (range 248–633) maternal deaths per 100,000 live births in Ghana for the same years (4). In 2011, Lozano and colleagues estimated 328 maternal deaths per 100,000 live births (range 247–409) with an annualized rate of decline of 0.9% between 1990 and 2011; they concluded that at the current pace Ghana is extremely unlikely to achieve a 75% reduction in maternal mortality by the year 2015, or even before 2040 [5]. Several interventions have been designed by governmental and non-governmental agencies, international, and national groups to curb this alarming situation in Ghana. The national health insurance scheme, which includes a free maternal health care package, is one such effort [18]. Nonetheless, highly vulnerable and hard to reach female sub-populations exist in the country [18,19]. One of these groups is the victims of unsafe abortion who must be identified for targeted interventions to occur. Abortion has been identified as one of the leading direct obstetric cause of maternal mortality in Ghana [20-22].

Previous studies in Ghana have identified young and single women as a high risk sub-population for abortion-related deaths [15,23]. The more we know about the characteristics of women at risk of abortion-related maternal mortality, the greater the chances of designing effective interventions and accelerate progress towards achieving MDG 5.

Ghana is a country with increasing alcohol consumption [24], a high number of unwanted pregnancies, and general disregard of contraceptive strategies [25,26]. A previous study has found that 12.9% of all adult women in Ghana consume alcohol, 4.4% are heavy drinkers, and 3.3% are risk-taking single-occasion drinkers [24]. Johnson and Madise, using data from 1998 and 2003, concluded that more than half of the women in the country are at risk of unwanted pregnancies [26]. Another study involving women admitted to hospital for post-abortion care found that only 17 percent had ever used contraception [25]. These behavioral characteristics heavily impact Ghana’s progress towards the achievement of MDG 5.

A lot of studies have found a positive association between alcohol consumption and spontaneous abortion. However, few studies conducted in high resource settings have investigated and found positive association between alcohol consumption and induced abortion. [27-29]. Such evidence is lacking in sub-Saharan Africa which bears the highest burden of abortion-related deaths and where access to and consumption of alcohol is less restricted. This study therefore aimed at investigating the possible correlation between alcohol consumption and induced-abortion among women who died from pregnancy related causes in Ghana. We hope this study will further elucidate the behavioral characteristics of victims of maternal deaths due to induced abortion and provide a possible pathway for targeted intervention toward reducing the mortality of childbearing women in Ghana.

## Method

### Data collection

The data for this study was extracted from the Ghana Maternal Health Survey 2007, which had been gathered by the Ghana Statistical Service in two phases. The sample covered 1600 clusters selected from the 10 administrative regions of Ghana across urban and rural areas. The primary sampling unit consisted of wards and sub-wards drawn from the 2001 population census. The sample size was estimated from the 2003 Demographic and Health Survey. In Phase I, 240,000 households were selected out of which 226, 209 completed the questionnaire. The questionnaire solicited the number of persons and deaths in a household by age and sex during the five years that preceded the survey. For female deaths, additional questions were posed, including whether the woman was pregnant at the time of death and whether she died during childbirth or within two months of delivery. The purpose of the questionnaire was to identify households for Phase II, the administration of the Verbal Autopsy Questionnaire. Households that had reported one or more deaths of women between the reproductive ages of 12 to 49 years in the five years that preceded the survey were revisited in Phase II to complete a verbal autopsy questionnaire. The verbal autopsy was used to identify true maternal deaths, defined as “the death of a woman during pregnancy or within 42 days of the end of pregnancy from causes related to or aggravated by the pregnancy or its management, but not from accidental or incidental causes” [30]*.* Verbal autopsy is an approach used to identify cause of death by interviewing lay respondents, usually close relatives, about the signs and symptoms experienced by the deceased before her demise [31]. It is employed in places with weak vital registration systems or where the availability of medical care is low [31].

**Figure 1:**
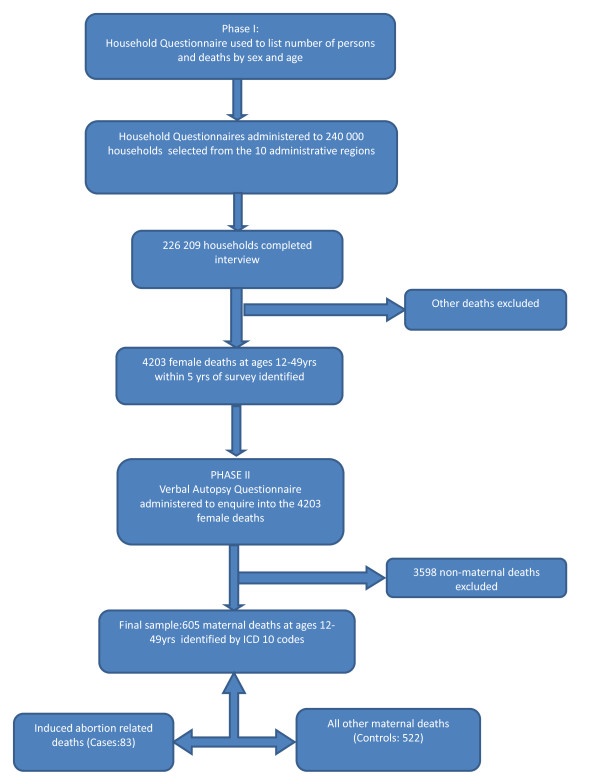
Flow chart on sample selection process.

The verbal autopsy questionnaire identified 4203 maternal and non-maternal female deaths in Phase II. The final causes of death were classified according to the ICD-10. Of the above total, 605 were maternal deaths in the 12 to 49-year-old age group, the sample that was used in this study. Figure 1 presents the sample selection process.

### Variables

The outcome variable was maternal deaths due to induced abortion. Induced abortion included medical, attempted, failed, unspecified, or other forms of abortion according to ICD-10 codes. All forms of spontaneous abortion (miscarriage) were excluded from the analysis.

The predictor variable was alcohol consumption patterns. Alcohol consumption patterns had four sub-variables: 1) *consumer of alcohol* (at any time in the past or present); 2) *consumer of alcohol within 12 months of death* 3) *frequency of alcohol consumption* (frequent consumer: consumed alcohol daily or weekly, occasional consumer: consumed alcohol once in a while, or abstainer: never consumed alcohol); and 4) *history of alcohol consumption* (less than one year, or one or more years).

### *Socio-demographic variables*

The socio-demographic variables used in this study were as follows:

 1. *Maternal age at death.* This variable was categorized into eight age groups (12–14, 15–19, 20–24, 25–29, 30–34, 35–39, 40–44, and 45–49) and further dichotomized into 18 and below, or over 18. We analyzed the eight narrow age groups and the two dichotomized ones.

 2. *Educational level*. This was classified in four categories.

 a. Never attended (women who confirmed having no formal education, and those whose educational levels were unknown)

 b. Basic education (women with some level of formal education not exceeding nine years, including those with primary, middle school, or lower secondary school education)

 c. Senior high school (women with up to 12 years of formal education or those whose education ended at the upper secondary school level)

 d. Tertiary or higher education (women who completed at least 15 years of formal education, including those with college, polytechnic, or university level studies)

 3. *Residence*. Residence was coded as either urban or rural.

 4. *Marital status*: Marital status was classified in two categories.

 a. Single (women who had never married, or were separated, divorced, or widowed at the time of death)

 b. Married (women who were married or living with a partner at the time of death).

### Measurement of variables, study design and statistical methods

In the first step of the analysis, we measured the prevalence of all the variables used in this study within the sample population. Logistic regression analysis was then carried out to examine the association between the different alcohol consumption patterns (predictor variables/covariates) and induced abortion (dependent variable). This could be described as a type of case control study design where the cases are maternal deaths from induced abortion and all the other maternal deaths served as the controls to which the cases were compared. The alcohol consumption variables used in the logistic regression analysis were: *ever consumed alcohol, and frequency of alcohol consumption.* The two outcome variables (*ever consumed alcohol and consumer of alcohol within 12 months of death*) were practically identical. All exposed and unexposed cases were the same and very few of the controls overlapped. Therefore, one could not expect the analyses to be but marginally different. So we chose to analyze only one of the two (*ever consumed alcohol*). Five logistic regression models were built. In model 1, the alcohol consumption variables were modeled on induced abortion to obtain the crude odds ratios of the different consumption patterns on induced abortion. In models 2 to 5, stepwise adjustments were made for maternal age, marital status, rural–urban residence status and educational level as potential confounders. These potential confounders were not included in the same regression analysis as this would most likely lead to an over-adjustment and falsely underestimate the impact of alcohol. Statistical software IBM SPSS Statistics 20 was used for the analysis.

## Results

Table 1 describes the characteristics of the samples. Among the 605 maternal deaths, 83(13.7%) died from induced abortion, 85 (14.3%) had ever consumed alcohol, 81 (13.6%) had consumed alcohol within 12 months of death. 25(4.2%) of the study population consumed alcohol frequently while 59(9.9%) were occasional consumers. A large number of those who consumed alcohol had been drinking for at least a year (89.6%). About 90% of the deaths occurred in women with low level of formal education (34.4% no education, 54.9% basic education). 44(7.3%) of the deaths occurred in women below 19 years of age, 99(16.4%) were single and 389(64.3%) resided in rural areas.

**Table 1:** Prevalence of induced abortion, other causes of maternal death, alcohol consumption, and socio-demographic characteristics among 605 women who died from pregnancy-related causes in Ghana between 2000 and 2005

**Variables**	**Number**	**%**
**Induced abortion**		
Yes	83	13.7
No	522	86.3
Haemorrhage	138	22.8
Hypertensive disorders of pregnancy	54	8.9
Sepsis	42	6.9
Obstructed labour	27	4.5
Miscarriage	20	3.3
Other infectious diseases^a^	84	13.9
Other non-infectious diseases^b^	75	12.4
Miscellaneous^c^	82	13.6
**Total**	**605**	**100**
**Ever Consumed alcohol**		
Yes	85	14.3
No	509	85.7
**Total**	**594**	**100**
Missing	11	
**Consumed alcohol within 12 months of death**		
Yes	81	13.6
No	513	86.4
**Total**	**594**	**100**
Missing	11	
**Level of alcohol consumption**		
Frequent consumer	25	4.2
Occasional consumer	59	9.9
Abstainer	510	85.9
**Total**	**594**	**100**
Missing	11	
**History of alcohol consumption**		
<Year	7	10.4^d^
≥Year	60	89.6^d^
**Educational level**		
Never attended	208	34.4
Basic Education	332	54.9
Senior secondary	52	8.6
Tertiary/Higher education	13	2.1
**Age**		
18 years and below	44	7.3
Above 18 years	561	92.7
**Age**		
12-14 years	3	0.5
15-19 years	62	10.2
20-24 years	115	19.0
25-29 years	133	22.0
30-34 years	116	19.2
35-39 years	106	17.5
40-44 years	55	9.1
45-49 years	15	2.5
**Residence**		
Rural	389	64.3
Urban	216	35.7
**Marital status**		
Single	99	16.4
Married	506	83.6

^a^ The major infectious diseases were malaria 53.6%, viral hepatitis 13.1%, unspecified infections 7.1% and tuberculosis 2.4%.

^b^ The major cause of death in the category of non-infectious diseases was Anaemia 41.3%; followed by diseases of blood and blood-forming organs 17.3%; respiratory diseases 14.6%; and circulatory diseases 12.0%.

^c^ Miscellaneous causes comprised mainly obstetric deaths of unspecified causes 26.8%, rupture of uterus 17.1%, complications of obstetric surgery 14.6%, embolism 9.8%, complications of anaesthesia 4.8% and other complications of pregnancy, labour and puerperium.

^d^ Only analyzed among individuals who drank alcohol.

Alcohol consumption was highest among women ages 30 to 34 years (25.9%), followed by 25 to 29 (24.7%), and 35 to 39 (15.3%). Women who had never attended school (49.4%) and those whose education ended at the basic level (44.7%) had the highest proportion of alcohol consumption, while those whose education ended at the secondary and tertiary levels had the least (4.7% and 1.2%, respectively). Rural residents showed the greatest percentage of alcohol consumption compared to urban residents. Among those who consumed alcohol and died from abortion, 92.3% had been drinking for at least one year. (not shown in Table).

Table 2 gives the crude odds ratio and 95% confidence interval between alcohol consumption and induced abortion. A significant association was found between alcohol consumption and deaths from induced abortion. Women who had ever consumed alcohol (OR _crude_ 1.9, 95% CI 1.04–3.35), frequent consumers (OR _crude_ 1.7, 95% CI 0.6–4.8) and occasional consumers (OR _crude_ 2.0, 95% CI 1.01–3.85) were about twice as likely to die from abortion related causes compared to those who abstained from alcohol.

**Table 2:** Association (Crude Odds ratios and 95% confidence intervals) between alcohol consumption and induced abortion among 605 women who died from pregnancy-related causes in Ghana between 2000 and 2005

**Variables**	**Induced Abortion**				
	**Yes**		**No**		**Crude OR(95% CI)**
	**n**	**%**	**n**	**%**	
**Ever Consumed alcohol**					
Yes	18	22.0	67	13.1	1.9 (1.04-3.35)
No	64	78.0	445	86.9	Ref
**Level of alcohol consumption**					
Frequent consumer	5	6.1	20	3.9	1.7 (0.63-4.8)
Occasional consumer	13	15.9	46	9.0	2.0 (1.01-3.85)
Abstainer	64	78.0	446	87.1	Ref

Table 3 gives the adjusted odds ratios and 95% confidence intervals of the association between alcohol consumption and induced abortion with a stepwise adjustment for maternal age, marital status, rural–urban residence status and educational level as potential confounders. Age, marital status and educational level were found to have a confounding effect on the observed association between alcohol consumption and induced abortion. Rural–urban residence status did not have any confounding effect on the association.

**Table 3:** Association (Adjusted Odds ratios and 95% confidence interval) between alcohol consumption and induced abortion among 605 women who died from pregnancy-related causes in Ghana between 2000 and 2005 with stepwise adjustment for potential confounders

**Variable**	**Model 1: OR (95% CI)**	**Model 2: OR (95% CI)**	**Model 3: OR (95% CI)**	**Model 4: OR (95% CI)**
**Ever Consumed alcohol**				
Yes	2.2(1.19-3.93)	2.4(1.29-4.44)	2.4(1.29-4.44)	2.6(1.38-4.87)
No	ref.	Ref.	Ref.	Ref.
**Level of alcohol consumption**				
Frequent consumer	2.1(0.74-5.73)	2.4(0.85-6.98)	2.4(0.86-7.08)	2.6(0.89-7.40)
Occasional consumer	2.3(1.15-4.47)	2.4(1.19-4.93)	2.4(1.19-4.90)	2.7(1.29-5.46)
Abstainer	Ref.	Ref.	Ref.	Ref.

Model 1: adjusted for age.

Model 2: adjusted for age and marital status.

Model 3: adjusted for age, marital status and rural/urban residence status.

Model 4: adjusted for age, marital status, rural/urban residence status and educational level.

The greatest confounding effect on the association between alcohol consumption and induced abortion was produced by age, followed by marital status and educational level. Those effects were very minor. Maternal age, marital status and educational level underestimated the above association. After adjusting for these confounders, women who had ever consumed alcohol (OR _adjusted_ 2.6, 95% CI 1.38–4.87), frequent consumers (OR _adjusted_ 2.6, 95% CI 0.89–7.40) and occasional consumers (OR _adjusted_ 2.7, 95% CI 1.29–5.46) were about three times as likely to die from abortion-related causes compared to those who abstained from alcohol.

The findings also revealed a rising trend in the risk of alcohol consumption with increasing age after adjusting for educational level, marital status and rural– urban residence status (OR _adjusted_ (95%CI) = 0.5*(0.28–0.81)*; 1.0 (*reference*);1.7(*1.20–2.36*); 2.0 (*1.43–2.75*); 2.4 (*1.75–3.45*), 2.5(*1.77–3.45*) and 2.9 (*2.05–4.09*))for ages 15–19, 20–24, 25–29, 30–34, 35–39, 40–44, and 45–49, respectively). There was also a statistically significant association between alcohol consumption and women who had never attended school (OR _adjusted_ 2.5, 95% CI = 1.28–5.01). A trend was also found between increased educational level and alcohol consumption, with increased education appearing to be a protective factor for the likelihood of alcohol consumption (never attended school OR _adjusted_ 2.5, basic education OR _adjusted_ 1.9, secondary education OR _adjusted_ 1.6, tertiary education OR _(ref)_ 1). No association was found between marital status, rural or urban residence, and alcohol consumption (not shown in Table).

## Discussion

This study found a positive association between alcohol consumption and the risk of maternal deaths from induced-abortion. It also revealed maternal age and educational level as possible factors lying behind the alcohol consumption patterns. Those who consumed alcohol were at more than twice the risk of dying from abortion than those who did not drink after adjusting for the effect of maternal age, marital status, rural–urban residence status and educational level (Table 3). We consider this of great importance for policy formulation and intervention design. Alcohol consumption was also found to have associations with age that were strong and increased as a person grew older.

This study supports previous studies from high resource settings that also found an association between alcohol consumption and deaths from induced abortion [32,33]. A study by Prager and colleagues found that alcohol abuse was significantly associated with increased odds of seeking repeat abortions [32]. Reardon and colleagues also reported that women who aborted reported more frequent use of alcohol when compared to those who carried the pregnancy to term [34]. A recent study on the socio-economic determinants of abortion rate by Gil-Lacruz and colleagues found that the alcohol consumption level for a region is an important lifestyle-related predictor of its abortion rate. They concluded from their study that higher alcohol prices reduces abortion rates and that raising the minimum drinking age is an effective policy for reducing levels of alcohol consumption and the number of unplanned pregnancies which ends up being aborted [28]. Sen also found in his study that alcohol taxes have statistically significant negative effects on teen abortion rates, and suggests that increased alcohol taxes may help prevent some unwanted pregnancies that would end up being terminated through induced abortion [27].

Previous researches have suggested possible pathways alcohol consumption could lead to increased risk of induced abortion and consequently death due to unsafe abortion. One possible pathway is that alcohol consumption causes impaired judgment which leads to increased risk of noncontracepted sexual intercourse and consequently abortion becomes an option as a result of unplanned or unwanted pregnancy [35,36]. Weinhardt and Carey in their review of literature concluded that there was lack of evidence that alcohol use causes unprotected sex although many of the studies they reviewed pertained to college students and adults [37]. Two years later, Sen from his study on the same association concluded that any alcohol use increases the probability of sexual intercourse and unprotected sex while heavy alcohol use generally has no effect [36]. This was consistent with findings by Ree and Colleagues [38]. Grossman and Markowitz improving on the weaknesses of aforementioned three studies, in his study concluded that risky sexual behaviours among teenagers were strongly correlated with alcohol consumption. They showed from their study that heavy drinking or drinking any amount all positively related to the probability of having sex and negatively related to the probabilities of using condoms or birth control [35]. Another study on the perceptions of contraception, the decision not to use protection and induced abortion among a sample of urban teenage girls identified alcohol consumption as the most cited determinant of risky sexual behaviours [39]. The impaired judgment caused by alcohol consumption directly affects the decision making power especially in young women [40]. Moreover, young women especially adolescents are ill-equipped to bear the responsibilities of unintended pregnancy and are therefore pressured into seeking abortion [41].

Another pathway that has been suggested by previous research is through the effect of alcohol myopia: where alcohol consumption leads to sufficient discounting of all future costs to the extent that all future consequences associated with pregnancy and its related outcomes are perceived as negligible [27]. Ghana has recently been listed among those countries with rising alcohol consumption [24]. This could eventually lead to an enormous problem especially among young women and those with low level of education who already bear a high risk of dying from induced abortion. A previous study from Ghana [15] found that the risk of dying from abortion decreases as maternal age increases. Earlier studies have also concluded that the risk of abortion-related deaths is greater among women under age 25 [15,42]. Although findings from previous studies suggest that the risk of dying from abortion is greatest in younger women, and we determined the risk of alcohol consumption to be greatest in older women, an association still appeared between alcohol consumption and abortion-related deaths. This may be explained by the influence alcohol consumption has on an individual’s decision making process. Although older women in Ghana are at the highest risk for excessive alcohol consumption, the detrimental effect of alcohol consumption on young women’s ability to make rational choices cannot be ruled out. Therefore, the influence of alcohol consumption on young women in Ghana is crucial and needs urgent attention.

Until 2008, Ghana had no governmental regulations concerning alcohol. In 2008, the first national alcohol policy for the country was drafted. Despite the ever-increasing trend toward alcohol consumption, the government’s effort to control alcohol consumption and reduce the harm to high risk populations has proved to be inadequate. Young women, especially adolescents must be prevented from exposure to unsafe abortions and the ensuing complications or death that may result. All of these measures will accelerate Ghana’s progress towards the achievement of MDG 5. Therefore, our study recommends that intense efforts should be made to increase the age at which alcohol may be legally consumed.

We also found alcohol consumption to be significantly associated with an individual’s educational level. Women who had no formal education constituted the highest risk group for alcohol consumption. Among those with some formal education, the risk of alcohol consumption decreased as their level of education increased. Thus, instruction in the responsible use of alcohol should not be limited to the educational institutions; targeted alcohol education should also be provided for those with no formal schooling. Since Ghana has not achieved universal basic education for all, it is essential that information on the health risks that alcohol and abortion pose be made available to communities in the informal sector with vulnerable sub-populations. Such an approach will contribute to reduce the growing inequity gap between the privileged and the less privileged in the country.

Although a large proportion of the women who consumed alcohol resided in rural areas, no association was found between rural residence and the risk of alcohol consumption. Therefore, the high prevalence of alcohol consumption that we observed in rural areas could be a reflection of the high proportion of the Ghanaian population living in such areas.

### Methodological consideration

To the best of our knowledge, this is the only study in Ghana and one of the few studies worldwide that has analyzed alcohol consumption patterns and deaths from induced abortion. A second strength is the representative nature of the study population. We utilized data from the first national population-based survey to collect information on maternal health and mortality in Ghana. Therefore, our results are generalizable to the entire Ghanaian population.

One limitation commonly encountered with the use of existing datasets is having to analyze the research question within the confines of the former. In this study, all the variables needed were available to us.

The use of deceased controls may be another issue of concern in this study. People who die, whatever the reason, tend to have a higher load of most risk factors for poor health than the general population. The most probable effect of using deceased controls in this study is that it will underestimate the impact of alcohol in the full population of pregnant women.

Another possible limitation was the use of verbal autopsy questionnaires. In Ghana, this procedure has been deemed highly sensitive and specific in identifying the causes of maternal deaths [43], although competent field interviewers are required in order to collect the information, and skilled office personnel are needed to assess, code, and analyze it. The technical expertise and resources available to the Ghana Statistical Service and its partners made this possible with minimal errors. Of the 5931 female deaths identified during the survey, verbal autopsy questionnaires were successfully completed for 4203 (70%). Due to the legal restrictions and the stigma faced by victims of induced abortion and their families in Ghana, it is possible that the non-response could be related to deaths from induced abortion. Also, there is likelihood for underreporting of alcohol consumption by the families involved. The fact that occasional consumption seems to be at least as risky as frequent consumption is counter-intuitive. The most probable explanation is that there is a larger proportion of non-participation/misclassification in the study of frequent consumers with abortion-related maternal deaths, compared with other types of maternal deaths, due to social stigma. These potential underreporting of both induced abortion related deaths and alcohol consumption could have underestimated the magnitude of the association found between alcohol consumption and deaths from induced abortion. Since potential non-differential misclassification of the cause of death and alcohol consumption was also a possibility, several steps were taken to minimize biases. First, induced abortion and other causes of maternal mortality were narrowly defined using specific ICD −10 codes. Information for some causes of death was cross-checked with death certificates, post mortem results, burial certificates, burial permits, antenatal cards, and hospital cards. Despite this effort, not all causes of death could be verified due to lack of data. It should be noted that deaths from miscarriage (included in our controls) has been linked to risky alcohol consumption in several studies, but this was not the case in our study since only 1 out of the 20 women who died from spontaneous abortion was reported to have ever consumed alcohol. Although regrettable, these biases were unlikely to affect the results of our study in the aggregate.

## Conclusion

Our findings reveal an important link between induced abortion-related deaths and alcohol consumption. We recommend further research to explore this association within the general Ghanaian population that includes all pregnant women. Policy actions directed toward reducing abortion-related deaths should consider alcohol consumption, especially among younger women. We suggest that information on the health risks posed by alcohol and abortion be disseminated to communities in the informal sector where vulnerable groups can best be reached. Efforts should also be made to increase the age at which alcohol may be legally consumed in Ghana.

## Competing interests

The authors declare that they have no competing interests.

## Authors' contributions

BOA was involved in the study design, data analysis and drafting of the manuscript. AA participated in the study design, data analysis and reviewed the manuscript. All authors read and approved the final manuscript.
